# Evaluating topological and graph-theoretical approaches to extract complex multimodal brain connectivity patterns in multiple sclerosis

**DOI:** 10.1007/s13755-025-00386-y

**Published:** 2025-10-19

**Authors:** Toni Lozano-Bagén, Eloy Martinez-Heras, Giuseppe Pontillo, Elisabeth Solana, Francesc Vivó, Maria Petracca, Alberto Calvi, Sandra Garrido-Romero, Albert Solé-Ribalta, Sara Llufriu, Ferran Prados, Jordi Casas-Roma

**Affiliations:** 1 Department of Computer Science, Universitat Autònoma de Barcelona, Barcelona, Spain; 2 Neuroimmunology and Multiple Sclerosis Unit, Laboratory of Advanced Imaging in Neuroimmunological Diseases (ImaginEM), Hospital Clínic de Barcelona, Institut d’Investigacions Biomédiques August Pi i Sunyer (IDIBAPS), Universitat de Barcelona, Barcelona, Spain; 3 Faculty of Brain Sciences, Department of Neuroinflammation, Queen Square MS Centre, UCL Institute of Neurology, University College London, London, United Kingdom; 4 Department of Neurosciences and Reproductive and Odontostomatological Sciences, University of Naples “Federico II”, Naples, Italy; 5 Department of Human Neurosciences, Sapienza University of Rome, Rome, Italy; 6 e-Health Center, Universitat Oberta de Catalunya, Barcelona, Spain; 7 Internet Interdisciplinary Institute (IN3), Universitat Oberta de Catalunya, Barcelona, Spain; 8 Department of Medical Physics and Biomedical Engineering, UCL Hawkes Institute, University College London, London, United Kingdom; 9 Facultad de Diseño, Innovación y Tecnología, Universidad de Diseño, Innovación y Tecnología (UDIT), Madrid, Spain; 10 Computer Vision Center, Universitat Autònoma de Barcelona, Barcelona, Spain

**Keywords:** MRI, Brain networks, Graph theory, Persistent homology, Multiple sclerosis, Machine learning

## Abstract

Brain networks, or graphs, derived from magnetic resonance imaging (MRI) offer a powerful framework for representing the structural, morphological, and functional organization of the brain. Graph-theoretical metrics have been widely employed to characterize properties such as efficiency, integration, and communication within these networks. More recently, topological data analysis techniques, such as persistent homology and Betti curves, have emerged as complementary approaches for capturing higher-order network patterns. In this study, we present a comparative analysis of these feature-generation methodologies in the context of neurodegenerative disease. Specifically, we evaluate the effectiveness of Betti curves and graph-theoretical metrics in extracting features for distinguishing people with multiple sclerosis (PwMS) from healthy volunteers (HV). Features are derived from structural connectivity, morphological gray matter, and resting-state functional networks, using both single layer and multilayer graph architectures. Our experiments, conducted on a cohort of PwMS and HV, demonstrate that features extracted using Betti curves generally outperform those based on graph-theoretical metrics. Furthermore, we show that multimodal data in terms of feature concatenation and multilayer graph architectures provide a more comprehensive representation of alterations in complex brain mechanisms associated with MS, leading to improved classification performance. These findings highlight the potential of topological features and multimodal integration for enhancing the understanding and diagnosis of neurodegenerative disorders.

## Introduction

Over the past decades, advances in magnetic resonance imaging (MRI) have greatly improved our capacity to investigate the structural and functional architecture of the human brain, enabling a deeper understanding of brain connectivity and functioning [1, 2]. The emergence of increasingly sophisticated pre-processing pipelines has further allowed for the integration of multiple imaging modalities, enabling the extraction of highly detailed information such as rich structural patterns in brain connectivity [3], the analysis of morphological characteristics of gray matter [4], and the mapping of functional brain networks [5]. These tools are up-to-date standards for exploring the brain’s complex system-level dynamics and have set the stage for more integrative and data-driven approaches to neuroscience [6, 7].

These structural patterns, morphological characteristics and functional brain networks–capturing some of the brain’s complex connectivity and behavioral dynamics–are commonly represented or studied as brain networks for its analysis. Brain networks provide a clear, robust, and effective mathematical framework for investigating both structural and functional properties [8–12]. This representation enables a wide range of interdisciplinary approaches: statistical physics offers tools to investigate global network organization and emergent dynamics [13, 14]; graph-theoretical frameworks allow quantification of topological properties such as modularity, centrality, and efficiency [11, 15–19]; and machine learning (ML) techniques are employed to extract predictive patterns and uncover latent network topologies [20–24, 24]. Among these approaches, ML stands out as particularly suited for predictive modeling, offering a flexible and scalable framework to address these complex challenges in neuroscience.

As in many other scientific domains, ML methods in neuroscience are designed to identify complex patterns by transforming data into numerical vectors or feature representations [25]. These multidimensional vector representations are fundamental for the subsequent application of classical predictive models, such as Support Vector Machines, Decision Trees, and Neural Networks, enabling classification and regression tasks. Thus, feature extraction becomes a crucial step in this process, allowing for the simplification of high-dimensional data (mainly image data or brain network representations) while preserving essential information. We can categorize the principal approaches in the literature into three main groups: vectorized network(graph)-theoretical metrics (VG), topological data analysis (TDA), and graph neural networks (GNNs).

The first approach, probably the most classical one [26], focuses on computing a variety of node- and network-level metrics–such as node degree, efficiency, and other centrality measures–to extract meaningful information from the graph’s topology [27]. These metrics are organized into feature vectors that capture key structural or functional properties of the network and its individual nodes, making them suitable for input into supervised ML models. This method has been widely adopted in neuroscience to analyze both structural and functional brain connectivity, and to investigate their relationships with cognitive function and clinical outcomes [3, 28–30]. We mainly identify applications to neurodegenerative disorders, such as Alzheimer [31, 32], closely related to brain loss of connectivity, and schizophrenia [33–35], but scholars have also explored neurocognitive [36] and psychiatric disorders [37, 38] with these tools. In this context, single network analysis is limited to a single characteristic or type of information, and hence, in some sense fails to fully describe the complexity of brain mechanisms and their interrelationship after damage. In order to overcome this limitation, a multilayer architecture–such as the one introduced in [39]–proves advantageous.

The second approach relates to topological data analysis (TDA) [40]. Algebraic topology is a field of mathematics that studies the qualitative properties of geometric spaces by representing them through algebraic structures. By translating features such as connectedness, loops, and voids into elements like groups or homology classes, it enables rigorous analysis and classification of spaces based on their underlying shape and structure, rather than their exact form or dimensions. One of the most widely used methods in TDA is persistent homology (PH), which builds on the concept of homology by tracking how features such as connected components, loops, and voids appear and disappear across different scales in a dataset. These filtered spaces (analogous to thresholded brain connectivity matrices) can be constructed from weighted graphs or point clouds, where edges between pairs of points are assigned weights based on a specific criterion, such as the distance between points or the strength of their connection. As the threshold on the edge weights changes, a sequence of nested structures emerges, capturing the evolution of topological features across different scales. In brain network analysis, the use of PH–first introduced in this context by [41] and typically applied to individual brain networks to capture the topological features of each subject’s brain network [42]–offers significant analytical advantages. This method is particularly useful in managing thresholding challenges involved in determining relevant connections within brain region interaction matrices, especially when dealing with functional interaction matrices. During the last decade, PH and several derived featurizations (e.g. Persistent Land-Scapes and Entropy, Carlson Coordinates and Betti curves), have been applied to study how brain connectivity relates to diseases and generative disorders, such as Alzheimer [43], Parkinson [44], Epilepsy [6], Schizophrenia [45] or, in general, to study brain processes [46, 47], such as perception [48], attention deficit hyperactivity disorder (ADHD) and autism spectrum disorder (ASD) [41]. For a comprehensive general overview of applying PH in ML tasks, see [49].

Finally, a third line of research has explored the use of graph neural networks (GNN’s) and related deep learning models to uncover patient-specific characteristics and disease-related patterns. These highly flexible, nonlinear architectures are expected to capture the intricate and complex interactions that characterize brain networks, with the aim of more effectively modeling their high-dimensional and richly structured connectivity. However, GNNs often face a major challenge: typical brain networks lack of rich node-level features (i.e., node embeddings). Because GNNs rely on the message-passing paradigm (or variation of it), generating suitable node embeddings becomes a crucial preprocessing step to enable effective learning and achieve strong performance. Recent approaches for constructing node features in brain graphs range from using Pearson correlation vectors to other ROIs as node embeddings [50], to incorporating information bottleneck strategies [51], or leveraging complex embeddings learned through advanced graph convolutional networks [52]. A comparative study of several of these methods is presented in [53], reporting classification performance of up to 73% accuracy for the task of Autism detection.

Each of the methods described above has its own advantages and drawbacks. At the featurization level, node-level centrality measures are highly informative and easy to interpret, since they are hand-crafted and grounded in clear logic. However, their main limitation is that they struggle to uncover unknown or unexpected patterns, as each measure is designed to capture a specific aspect of the network and its repercussions on the overall graph. Persistent homology provides a natural framework for studying the intrinsic structure of a graph. However, its ability to capture the underlying dynamics is more limited compared to graph-theoretic features. Finally, GNNs are highly flexible and capable of learning very complex patterns, but their interpretability is restricted, making it difficult to fully understand which aspects of the network are being learned. Analyzing the different data modalities and their combinations, it becomes clear that multimodal data offers a richer and more informative framework, albeit at a higher computational cost. Therefore, it is important to assess whether the benefits gained from multimodal integration truly outweigh the additional complexity.

The primary objective of this study is to analyze and evaluate the discriminative capability of graph-theoretical metrics, TDA, and GNN-based methods by employing features derived from single and multimodal characterization of brain networks. Specifically, we extract topological features using graph-theoretical metrics and Betti curves from three types of brain networks: (1) structural connectivity (diffusion MRI), (2) morphological gray matter networks (T1-weighted MRI), and (3) functional networks (resting-state fMRI). We perform both unimodal and multimodal analyses to assess the individual and combined contributions of these modalities. Finally, we evaluate the classification performance of the proposed methodology in distinguishing people with multiple sclerosis (PwMS)–a chronic, inflammatory, demyelinating, and neurodegenerative disease of the central nervous system characterized by widespread tissue damage that disrupts both large- and short-scale structural and functional connectivity, ultimately leading to clinical impairment [54, 55]–from healthy volunteers (HV) [56], in order to determine which network signatures best capture the underlying pathophysiology of multiple sclerosis. Therefore, our study also contributes a systematic comparison of the effectiveness of traditional graph-theoretical descriptors, topological data analysis, and graph neural networks in identifying meaningful alterations in brain organization associated with MS. Ultimately, this work supports the development of more accurate automated predictors and monitoring tools for multiple sclerosis.

We believe that addressing these questions and examining which data modalities–or their combinations–are most effective for predictive tasks, how different ML algorithms perform, and how their features can be modeled, can provide useful guidance for researchers studying brain networks and their relationship to brain disease. By outlining the strengths and limitations of different approaches, our work seeks to contribute to more robust methodologies and support future studies in this field.

## Materials and methods

This study uses data extracted from patients diagnosed with MS according to the McDonald 2017 criteria [57] and HV from the radiological and clinical databases of the MS Center of the University of Naples “Federico II”. The project was carried out in accordance with the declaration of Helsinki, with written informed consent obtained from each participant.

### Participants

Among a cohort of $n=52$ PwMS (35 women) analyzed, 30 had relapsing-remitting MS, 7 had primary MS and 15 had secondary progressive MS. The mean age was $42.50 \pm 12.80$ years, mean disease duration was $13.10 \pm 9.05$ years, and the median EDSS (Expanded Disability Status Scale) was 4.5 (range 1.5$-$7.0). A group of $n=53$ health volunteers (HV) (33 women), with a mean age of $41.30 \pm 11.63$ years was used as a reference group. Table 1 summarizes the clinical and demographic data of the cohort.

**Table 1:** Clinical and demographic data. Continuous variables are given as the mean ± standard deviation. EDSS stands for the *Expanded Disability Status Scale* and *p* values obtained from comparing the groups

	Healthy volunteers	People with MS	*p* value
Number of subjects, *n*	53	52	–
Age, years	$41.30 \pm 11.63$	$42.50 \pm 12.80$	$<0.001$
Female, *n* (%)	33 (62.26%)	35 (67.31%)	$<0.001$
Disease duration, years	–	$13.10 \pm 9.05$	–
Median EDSS score (range)	–	4.5 (1.5-7.0)	–

### Magnetic resonance acquisition details

MRI acquisition protocols were conducted using a 3T Magnetom Trio scanner (SIEMENS, Erlanger, Germany) equipped with an 8-channel phased-array head coil. The acquisition included high-resolution three-dimensional Magnetization-Prepared Rapid Acquisition with Gradient Echo (3D-MPRAGE) images, with the following parameters: TR = 3000 ms; TE = 24.1 ms; TI = 1000 ms; 224 sagittal slices; 0.80 mm isotropic voxel size; and a matrix size of $320 \times 320$. Additionally, three-dimensional Fluid Attenuated Inversion Recovery (3D-T2 FLAIR) images were obtained with the following parameters: TR=6000 ms; TE=404 ms; TI = 2200 ms; 160 sagittal slices; 1.00 mm isotropic voxel size; and a matrix size of $256 \times 256$. The Diffusion-Weighted Imaging (DWI) protocol featured: TR = 7400 ms; TE = 88 ms; 60 contiguous axial slices; 2.20 mm isotropic voxel size; a matrix size of $104 \times 104$; a b value of 1000 s/$\hbox {mm}^2$; 64 diffusion encoding directions; and 8 images acquired at 0 s/$\hbox {mm}^2$ randomly interspersed throughout the acquisition. For all subjects, the same resting-state functional MRI (RS-fMRI) protocol was acquired, employing a BOLD EPI pulse sequence with fat saturation. The parameters were as follows: TR = 2500 ms; TE = 40 ms; 30 axial slices with a 1 mm gap between slices; a voxel size of $3 \times 3 \times 4$ $mm^{3}$; a matrix size of $64 \times 64$; and a total of 200 frames.

### Multimodal processing pipelines

The complete processing pipeline of the different modalities of MRI data can be found in [39]. White Matter (WM) lesions were detected using 3D-MPRAGE and 3D-FLAIR imaging techniques to accurately segment the 76 gray matter (GM) regions from “filled” 3D- MPRAGE images [58]. This facilitated the development of an anatomical scheme for the networks [59]. For the morphological network, GM segmentations were re-sliced to 2 mm isotropic voxels, and regional morphological similarity was calculated by averaging the Pearson’s correlation coefficients between neighboring $3 \times 3 \times 5$ voxel cubes within each of the 76 parcellated brain regions, using the methodology described by [4]. For the structural brain connectivity network, the DWI preprocessing pipeline included MP-PCA denoising, Gibbs ringing removal, and correction for eddy currents and motion. Fractional anisotropy (FA) maps were then computed using FSL’s DTIFIT [60]. Probabilistic streamline tractography, incorporating an Anatomically Constrained Tractography (ACT) framework, was used to generate FA-weighted connectivity matrices [61]. For the functional brain network, the RS-fMRI data underwent a standard preprocessing pipeline in FSL, including slice timing and motion correction, spatial normalization, and band-pass filtering (0.001$-$0.08 Hz) [62]. The absolute values of the correlations between the average time series for each pair of the 76 brain regions were used to construct the functional connectivity matrices. For all three modalities, networks were adjusted for age and gender effects using linear regression, where age and gender were included as covariates. The corrected network values correspond to the model residuals centered at the mean.

The GM, DTI, and RS-fMRI networks were derived from 3 MRI modalities acquired during the same scan session using a 3T Siemens scan. Therefore, each subject is modelled by three single layer networks representing GM morphology, DTI structural connectivity, and RS-fMRI functional activity. The 76 nodes of the three networks represent the common anatomical parcellation scheme (i.e., nodes of all networks are equivalent and represent the same anatomical brain region), and edges represent the morphology, structural connectivity, or functional values between each pair of nodes.

#### Morphological gray matter networks

The construction of the GM morphological network relies on the comparison of GM morphological patterns as delineated by the specified anatomical parcellation scheme [4]. The nodes of the GM network correspond to 76 distinct anatomical regions, while the edges between nodes represent similarities or correlations in the morphological features of the GM (e.g., volume, thickness, surface area). The values of GM morphological matrices are in the range [0, 1].

#### Structural brain connectivity networks

The DTI structural connectivity involved aligning the parcellation scheme (comprising 76 nodes) from the anatomical image to the fractional anisotropy (FA) map, making it easier to identify the streamline connections needed between node pairs to construct the structural connectome. For each tract connecting different brain nodes, the FA values, which range from 0 to 1 for each voxel along the tract, were averaged to generate the FA-weighted adjacency matrix for the network. By computing the mean FA along the fiber pathways linking brain regions, we encapsulated the severity of WM damage at both macro- and micro-structural levels [63]. Each edge in the resulting network layer has values ranging from 0 to 1.

#### Functional brain networks

The RS-fMRI matrix, capturing brain signal correlation and synchronization, was acquired according to the methodology outlined by [62]. Following several pre-processing and post-processing steps [39], the pairwise signal Pearson’s correlations were assessed from the 76 brain regions. These correlation values range from −1 to 1, indicating either negative or positive relationships between nodes. However, to focus solely on the strength of these relationships, irrespective of their direction, we apply the absolute value to these correlations.

#### Multilayer brain networks

**Fig. 1:**
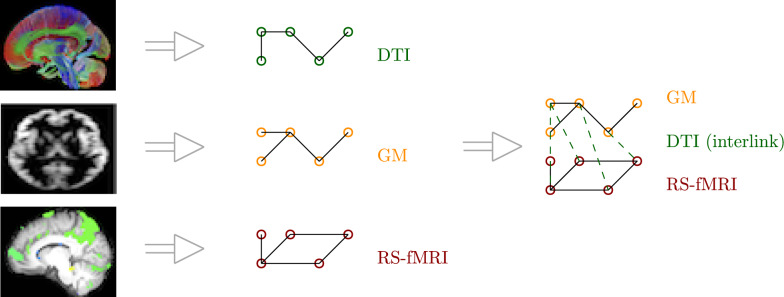
Scheme to create the single layers networks (center) and the multilayer network (right), as defined in [39]

Finally, our comparison included the multilayer scheme developed in [39], as a complex network composed of different layers, each representing a single type of relationship between nodes within one layer. In this multilayer network, each subject is represented by three single layer networks representing GM morphology, DTI structural connectivity, and RS-fMRI functional activity, which are combined to create a multilayer network composed of two layers, as can be seen in Figure 1. Nodes represent the same exact object in each of the different layers, and encode different types of relationships throughout their edges. In this type of network, we differentiate between intralayer links, which encode the single type of relationship the layer represents, and interlayer links, which encode how the different node perspectives (types of relationships) are related within the system.

Following previous research [39], the authors proposed encoding GM and RS-fMRI within the layers of the multilayer graph, while using DTI for interlayer links. DTI interlinks provide insights into the structural connectivity of the brain by mapping the WM tracts that physically connect different cortical regions. This approach was chosen because DTI structural connectivity matrices represent the integrity of WM fiber pathways between GM brain regions, with values ranging from 0 (isotropic diffusivity) and 1 (anisotropic diffusivity). These tracts serve as the “roads” over which neural signals travel, supporting functional communication. GM regions are the hubs of neuronal activity, where computations occur, while RS-fMRI measures the blood-oxygen-level-dependent (BOLD) signal, reflecting neuronal activity. By linking these two, the multilayer model aims to correlate the structural “roads” with the functional “traffic”. This natural relationship between brain structure and function helps to build a more biologically informed and interpretable multilayer model.

#### Networks representations

For each subject in the dataset, we have assessed four possibilities to represent their brain state and structure:

- Single layer graph with DTI structural connectivity network.
- Single layer graph with GM morphological network.
- Single layer graph with RS-fMRI functional network.
- Multilayer graph with GM, DTI and RS-fMRI network, following the architecture previously described.

### Methodology

To measure the predictive power of the different featurizations as well as the difference between single and multilayer representations (detailed in Section 2.3) to distinguish people with MS from healthy volunteers, we employed two different feature extraction techniques and applied multiple ML models, as described below.

#### Feature extraction

This section describes the feature extraction techniques, a crucial step in training machine learning models, as it involves representing each graph using a fixed set of numerical descriptors. To this end, we propose the use of two complementary approaches. First, we present the technique based on persistent homology and Betti curves, which captures topological properties of the graphs. Second, we introduce a set of graph-theoretical metrics that characterize structural aspects of the graphs.

#### Persistent homology and betti curves

PH and Betti curves are powerful tools within the field of TDA, offering several advantageous properties for analyzing complex data structures [64]. PH enables the examination of topological features (such as connected components, loops, and voids) across multiple scales, providing a rich multiscale representation of the data’s structure [65]. This is particularly valuable when analyzing brain networks, where features of interest may manifest at different levels of granularity. The process starts by considering data as points in a space (e.g., a point cloud), but instead of focusing only on the individual points, it studies how these points connect and form shapes, such as clusters, loops, or voids. To capture the structure of the data, PH examines it at different scales by constructing a sequence of shapes (called a filtration). In particular, it starts with small neighborhoods around points and gradually increases their size to observe how features like connected components, loops, or voids appear and disappear. More persistent features are detected over a wide range of spatial scales and are deemed more likely to represent true features of the underlying space rather than artifacts of sampling, noise, or particular choice of parameters. This enables us to measure how the construction and structure of the graph impact the model’s outcomes, while isolating any other information unrelated to the topology.

One of the principal strengths of PH and Betti curves is their robustness to noise. Topological features that persist across a wide range of the filtration parameter are likely to correspond to meaningful structures in the data, while short-lived features are typically considered noise. Moreover, PH facilitates dimensionality reduction by emphasizing essential topological features, allowing for more interpretable analyses of high-dimensional neuroimaging data. Betti curves, in particular, offer a compact and intuitive summary of this information by tracking the number of topological features (e.g., Betti-0: components; Betti-1: loops; Betti-2: voids) as a function of scale. This concise representation makes Betti curves especially suitable for integration into supervised ML pipelines.

In TDA, each shape in the filtration sequence is often represented as a simplicial complex, which consists of vertices, edges, triangles, and higher-dimensional analogs. These simplicial complexes can also be constructed from weighted and undirected graphs, such as those constructed from brain connectivity measures. The weights are defined as detailed in Section 2.3 and all values are in range [0,1], where higher values indicate stronger connectivity or correlation scores. However, considering that PH typically associates tighter connections with lower values, it was necessary to adjust the edge information accordingly. Therefore, to adapt brain connectivity graphs to the implementation of the Giotto TDA library^1^ used in this work, all edge weights were transformed by computing its inverse. Among the various available options, we opted to invert the edge weights using the following equation:

1 $$\begin{aligned} \widehat{e}_{ij} = 1 - e_{ij} \end{aligned}$$

where $e_{ij} \in E$ denote an undirected edge between nodes $v_i \in V$ and $v_j \in V$ in the graph *G*(*V*, *E*), which corresponds to the entry (*i*, *j*) in the adjacency matrix *A*. We denote by $\widehat{e}_{ij}$ the same edge when computed using the inverse score.

**Fig. 2:**
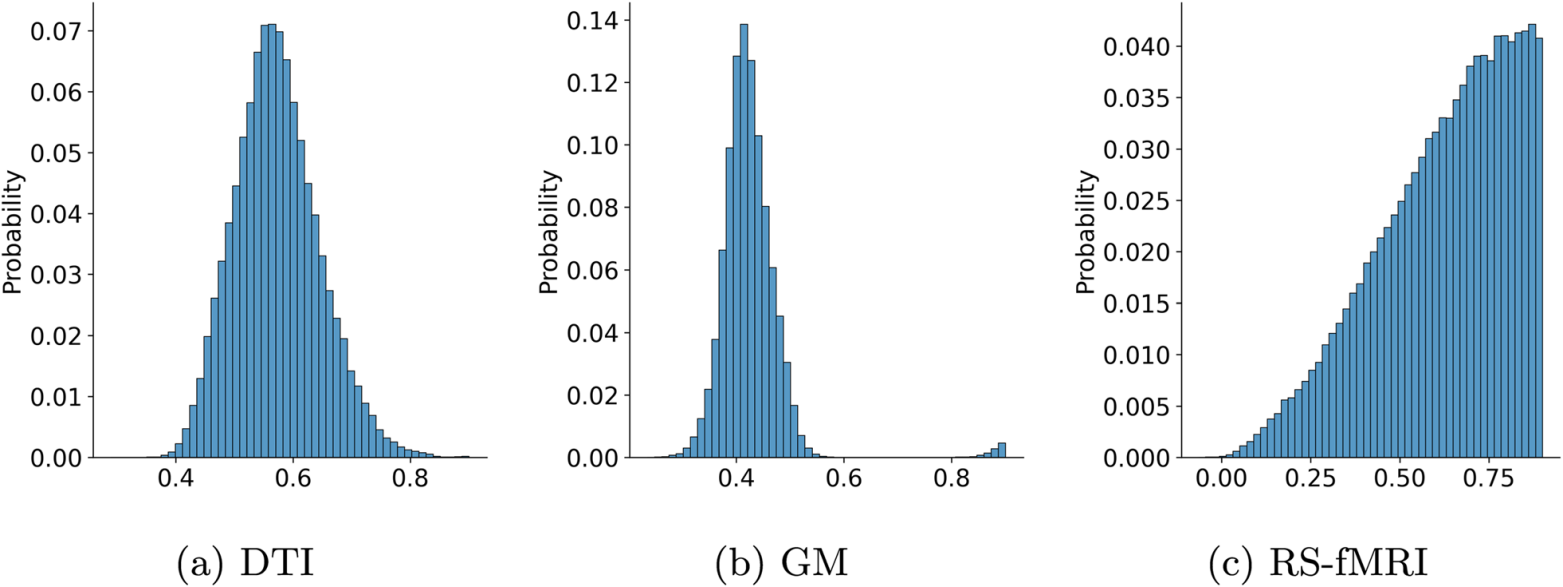
Histogram of the inverse edge weights for DTI, GM, and RS-fMRI values, computed using Equation 1. The histograms were generated using data from all individuals in our dataset, including both people with MS and healthy volunteers

The histograms of $\widehat{e}_{ij}$ for the different single layer graphs are presented in Figure 2, providing deeper insight into the data and a better understanding of the value distribution for each matrix type (DTI, GM and RS-fMRI). DTI values are predominantly distributed within the range of 0.4 to 0.8, following a normal distribution. In contrast, GM values exhibit a similar pattern but with a narrower distribution, primarily concentrated between 0.3 and 0.5. The correlation values of RS-fMRI span the entire range between 0 and 1 but are predominantly concentrated in the higher range of the histogram when considering the inverse values. This indicates that, in the original matrices, these values are primarily concentrated in the lower range values.

After computing the inverse edge weights for each matrix type, a nested sequence of simplicial complexes is generated by varying a filtration parameter (threshold) from 0 to 1. PH tracks the appearance and disappearance of topological features across this filtration.

The result is often visualized as a persistence diagram or barcode, which encodes the lifespan of features as intervals (*b*, *d*), where *b* is the birth and *d* is the death of a feature. The PH diagrams have a variable number of features for each subject, depending on the actual topological structure of each graph and the dimensions used when computing homology. Although PH can be computed in any dimension, its computational cost increases significantly with higher dimensions, as the number of simplices grows exponentially. Three experiments were conducted in this study: the first involving dimension 0; the second involving dimensions 0 and 1; and the third one using dimensions 0, 1, and 2. Experiments conducted considering more than three dimensions but did not offer additional insights and involve high computational complexity, so we did not include them in this study. This approach enabled us to validate whether PH yields consistent results when different dimensions are employed, thus highlighting that its main advantage derives from the structure of the data.

**Fig. 3:**
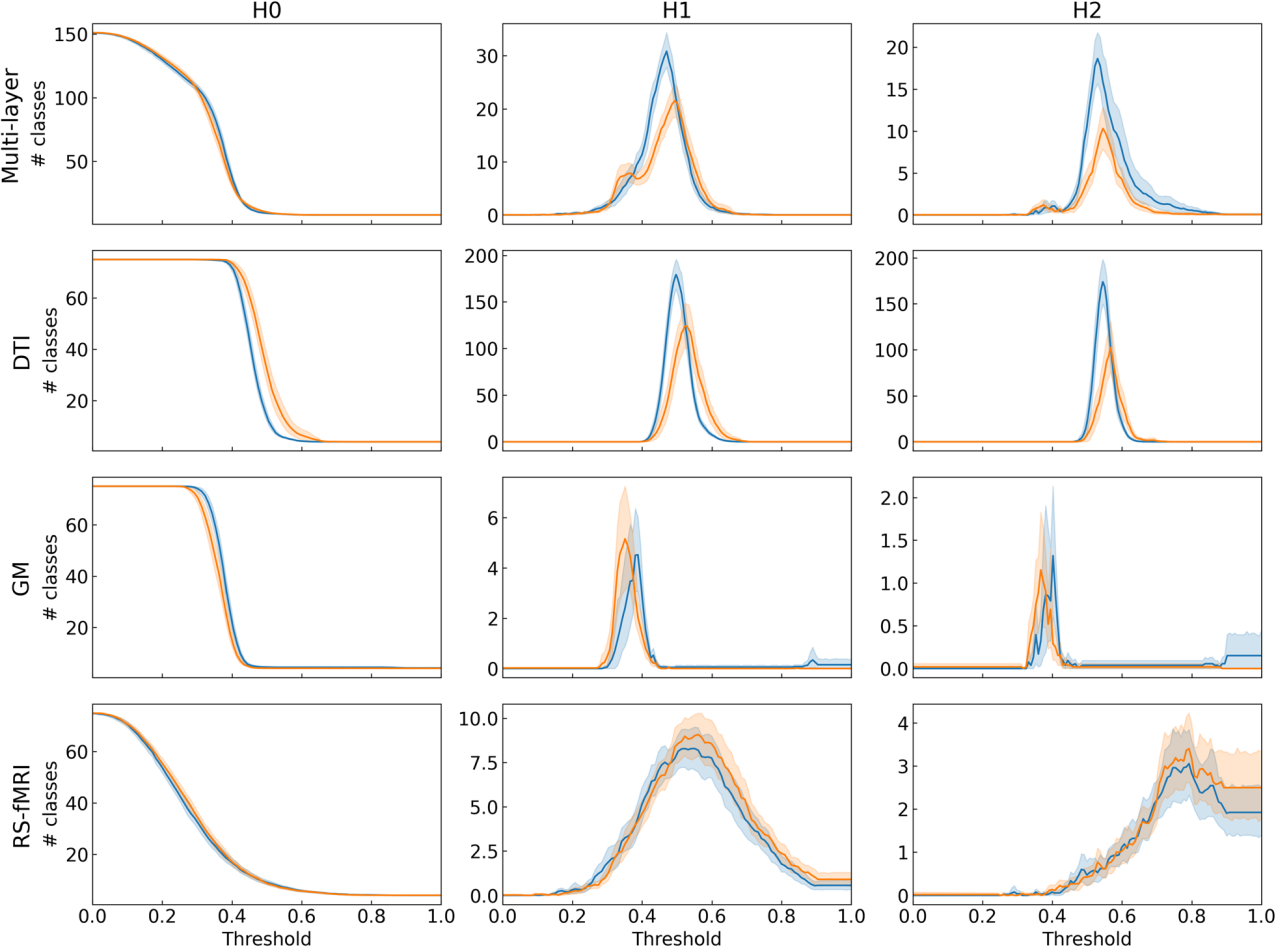
The average and standard deviation of the Betti curves are presented for each group, with HV represented in blue and PwMS in orange

To better understand the information captured by this approach, Figure 3 displays the mean and standard deviation of the Betti curves for each group. The curves for HV and PwMS are shown separately to highlight differences in their topological characteristics.

In these diagrams, each curve represents a dimension, and each point in the curve represents how many topological features are alive for each possible value of the filtration parameter. By construction, we can also see in these diagrams that the structure of topological features is different in each type of brain network and also between patients and control groups, so the ML models should be able to react differently to the different graphs using the features extracted from the Betti curves.

In the case of multilayer networks, it is important to note that we use the super-adjacency matrix [66] to compute PH. The use of the super-adjacency matrix doubles the number of nodes, resulting in 2*N* nodes (where *N* is the number of nodes in the single layer graphs). However, this increase does not affect the results of the computation, as PH can be computed independently of the number of nodes.

To use PH information in a traditional ML model, the variable number of topological features need to be converted into a fixed number of numerical features. Various methods can be employed to convert a persistent diagram with a variable number of features to a vector with a fixed number of components. In this case, we propose to use Betti curves [67], which have proven to be a powerful tool for enhancing time series analysis when combined with ML. They have demonstrated robustness and effectiveness, particularly in classification tasks [68].

Betti numbers are fundamental topological invariants that count the number of *k*-dimensional holes in a topological space, where $\beta _0$ is the number of connected components, $\beta _1$ is the number of loops (1-dimensional holes), $\beta _2$ is the number of voids (2-dimensional holes), and so on. Betti curves are derived from PH and represent how Betti numbers vary across different values for the filtration parameter. Specifically, a Betti curve is a plot of Betti numbers ($\beta _k$) as a function of the filtration parameter, providing a simplified summary of the information captured by PH. Thus, Betti curves show the total count of features for each dimension at a given scale [42].

#### Graph-theoretical metrics

We employ five graph-theoretical metrics [69] to characterize individual nodes, which are then combined and utilized as features for each graph. These metrics are defined as follows:

- *Degree*: One of the most straightforward and widely utilized centrality metrics, degree is frequently applied in social network analysis to evaluate node significance. It is defined as the number of edges connected to a specific node and can be viewed as a simplified counterpart to node strength.
- *Strength*: This metric extends the concept of degree by incorporating edge weights. It is calculated as the sum of the weights of all edges incident to a node and acts as a foundational indicator of a node’s importance in weighted networks.
- *Local efficiency*: Efficiency measures the effectiveness of information transfer within a network [70]. Local efficiency specifically assesses the communication efficiency among a node’s immediate neighbors, indicating the network’s robustness against localized disruptions. In brain networks, both local and global efficiency have been linked to cognitive processes such as working memory and functional integration [71].
- *Betweenness centrality*: This measure quantifies the frequency with which a node lies on the shortest paths between other nodes, capturing a distinct perspective on node significance. Unlike degree and strength, betweenness centrality is a global metric that reflects a node’s potential to influence information flow across the network.
- *Closeness centrality*: Closeness centrality evaluates the average shortest path distance from a given node to all other reachable nodes in the network. It provides insight into the “spatial” position of nodes in the network and, similar to betweenness, serves as a global metric for describing the network.

The application of these metrics within the multilayer framework is explored and elaborated upon in the study by [39]. We refer the reader to such work for details on how to compute this measures on multilayer setups.

#### Experimental framework

**Fig. 4:**
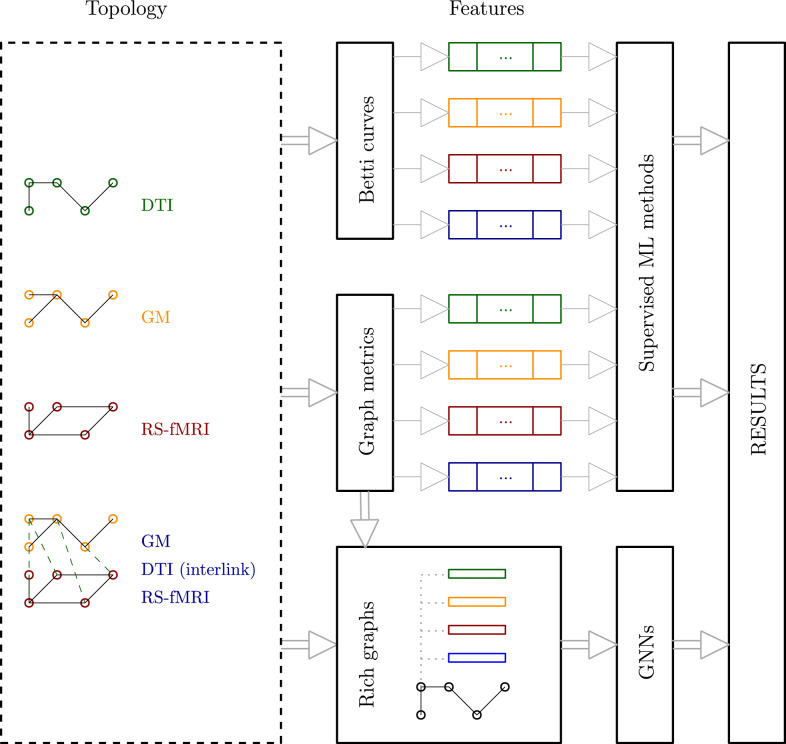
Pipeline designed for the experimental framework

We propose three distinct pipelines for predicting outcomes from brain networks, each defined by a different feature extraction strategy. All three pipelines follow a common structure that includes the key processing steps required to extract and transform information from the brain networks into predictive features, as depicted in Figure 4.

The *first pipeline* is based on PH and Betti curves, leveraging their capacity to capture topological features of a space across multiple spatial resolutions. In the initial step, persistent diagrams–generated through PH–are transformed into fixed-size vectors using Betti curves. Specifically, we evaluate the Betti numbers over 100 equally spaced values of the filtration parameter in the interval [0,1], resulting in a fixed-length vector for each topological dimension considered. These vectors are then concatenated and used as inputs to train supervised ML models. To account for the multilayer architecture, which simultaneously incorporates all types of connectivity, we also concatenate the feature vectors derived from the three corresponding single layer graphs before training the models.

The *second pipeline* follows a similar supervised learning approach but substitutes topological features with graph-theoretical metrics. This pipeline involves a two-step process: first, we compute various graph-theoretical metrics for each node in the graph; second, for each metric, we concatenate the values across all nodes to form a feature vector representing the entire graph. These vectors are then used to train supervised ML models. Similarly to the previous pipeline, we concatenate the feature vectors obtained from the three single-layer graphs to enable a fair comparison with the multilayer architecture, which integrates all connectivity types within a unified framework. In addition, we conduct an extra experiment in which all graph-theoretical metrics are combined into a single feature vector for each single layer, effectively creating a multi-feature configuration for each single-layer network. This configuration is evaluated from three perspectives. First, we train the models using the complete feature vector. However, given the relatively high dimensionality of this vector (five features per node, i.e., $5 \times 76 = 380$ features), we also assess two strategies for dimensionality reduction and feature selection. For dimensionality reduction, we applied Principal Component Analysis (PCA) and retained 20 components, which, based on a parameter grid search, captured approximately 80% of the explained variance. For feature selection, we leveraged feature importance scores derived from a Random Forest model trained with 100 estimators and the Gini impurity criterion, selecting the top 100 most important features, which preserved roughly 80% of the impurity-based importance.

The *third pipeline* diverges from the previous two by utilizing GNNs and embedding graph-theoretical metrics as node features. Since the brain networks under study lack inherent node attributes, we enrich the graphs (whether based on a single modality or a multilayer structure) by embedding graph-theoretical features at the node level. We explore five GNN architectures: Graph Convolutional Network (GCN), Graph Attention Network (GAT), Graph Isomorphism Network (GIN), Chebyshev Graph Convolutional Neural Network (ChebGCN) and GraphSAGE. In this setting, the node features are derived from the graph-theoretical metrics computed specifically for each brain representation. The graph-theoretical metrics employed are the same as those used in the previous pipelines, but adapted to the multilayer architecture (details of their definition and computation can be found in [39]). Additionally, we conduct a multimodal experiment in which the single layer DTI graph structure is augmented with node features extracted from GM and RS-fMRI modalities. This approach enables the GNN to integrate heterogeneous information from multiple layers of brain connectivity.

#### Machine learning models

To evaluate the effectiveness of features extracted using Betti curves and graph-theoretical metrics, different supervised ML models are trained on each type of architecture to distinguish between PwMS and HV. To test the generalizability and stability of each chosen architecture, the same experiment is repeated 10 times with random initializations for the subjects within each fold of a 4-fold cross-validation strategy. This approach helps mitigate overfitting and makes efficient use of the limited data available by ensuring that all samples are used for both training and validation across different folds. As our primary evaluation metric, we use the Area Under the Receiver Operating Characteristic Curve (AUC-ROC), which is computed from the predicted scores and is particularly appropriate given our dataset. AUC-ROC provides a robust measure by evaluating the model’s ability to discriminate between classes across all possible classification thresholds.

We consider both traditional supervised ML models and GNNs for the classification task between PwMS and HV. For the traditional supervised ML models, we evaluate the following approaches:

- A Fully Connected *Neural Network* (NN) with 3 hidden layers of 200, 100 and 10 neurons, with ReLU activation function.
- A *Logistic regression* (LR) with L2 penalty.
- A *Random Forest* classifier (RF) with 100 fully grown trees.
- A *Support Vector Machine* (SVM) with an RBF kernel, scaled gamma and regularization parameter $C=1.0$.

Regarding the use of GNNs, we compare several architectural designs and conduct a grid search to identify the optimal hyperparameters. Specifically, we evaluate the following graph neural network architectures:

- *Graph Convolutional Network* (GCN) introduced by Kipf and Welling [72]. We test two configurations: (i) a single-layer architecture consisting of a convolutional operator with 32 hidden units, followed by ReLU activation, max pooling, and a final linear layer; and (ii) a two-layer architecture, where the first layer is identical to the single-layer setup, while the second layer applies the same operator with 16 hidden units, followed by max pooling and a linear layer.
- *Graph Attention Network* (GAT) proposed by Veličković et al. [73]. We adopt the same two configurations as for the GCN, but replace the convolutional operator with an attention-based operator and use ELU activation. Each layer employs four attention heads, and a dropout probability of 0.6 is applied to the normalized attention coefficients.
- *Graph Isomorphism Network* (GIN) [74], a representative model in the message-passing paradigm based on the Weisfeiler–Lehman graph isomorphism test. We evaluate two- and three-layer configurations, where each layer comprises a linear transformation, batch normalization, ReLU activation, another linear transformation with ReLU activation, add pooling, and dropout with a probability of 0.5, followed by a final linear layer.
- *Chebyshev Graph Convolutional Neural Network* (ChebGCN) [75], a spectral graph convolution approach that leverages Chebyshev polynomials to design fast, localized filters. Unlike spatial GNNs, ChebGCNs are based on spectral formulations derived from graph theory. We evaluate a two-layer architecture using Chebyshev filters of size 3 and 5 with symmetric normalization, followed by a linear layer with ReLU activation, dropout (0.5), and a global max pooling operator.
- *GraphSAGE* [76], an inductive framework that exploits node feature information. We consider an architecture with three GraphSAGE layers, each followed by ReLU activation, a global mean pooling operator, a linear layer with ReLU activation and dropout (0.5), and a final linear layer.

For all configurations, we employ the Adam optimizer with cross-entropy loss. The grid search is conducted over the following hyperparameters: learning rate $\{0.01, 0.001, 0.0001\}$ and weight decay $\{5 \times 10^{-4}, 5 \times 10^{-6}\}$.

## Results

Regarding the *first pipeline*, which employs Betti curves for feature extraction and supervised ML models to classify subjects as either HV or PwMS, Table 2 summarizes the results of the experiments described for homology dimensions 0, 1, and 2, incrementally incorporating higher-dimensional homologies.

**Table 2:** AUC ROC values (mean ± std) for different ML models applied to the different processing of the brain connectivity data. Results are reported using features extracted from homology dimensions 0; 0 and 1; and 0, 1, and 2. NN denotes Neural Network, LR Logistic Regression, RF Random Forest, and SVM Support Vector Machine. The last column shows the average across all models, with bold values indicating average performance $\ge 0.70$

Homology	Network architecture	NN	LR	RF	SVM	Average
dimensions						
0, 1 and 2	Single layer DTI	$0.71 \pm 0.03$	$0.73 \pm 0.03$	$0.81 \pm 0.01$	$0.77 \pm 0.01$	**0.76** ± 0.04
	Single layer GM	$0.68 \pm 0.03$	$0.64 \pm 0.03$	$0.73 \pm 0.20$	$0.65 \pm 0.02$	$0.68 \pm 0.03$
	Single layer RS-fMRI	$0.43 \pm 0.03$	$0.43 \pm 0.03$	$0.48 \pm 0.04$	$0.49 \pm 0.02$	$0.44 \pm 0.05$
	Concatenation of SL	$0.75 \pm 0.01$	$0.74 \pm 0.01$	$0.81 \pm 0.01$	$0.73 \pm 0.02$	**0.75** $\pm 0.03$
	Multilayer	$0.81 \pm 0.02$	$0.81 \pm 0.01$	$0.83 \pm 0.01$	$0.78 \pm 0.02$	**0.80** $\pm 0.02$
0 and 1	Single layer DTI	$0.71 \pm 0.02$	$0.75 \pm 0.01$	$0.80 \pm 0.01$	$0.75 \pm 0.01$	**0.76** $\pm 0.04$
	Single layer GM	$0.70 \pm 0.02$	$0.66 \pm 0.02$	$0.73 \pm 0.02$	$0.61 \pm 0.03$	$0.67 \pm 0.05$
	Single layer RS-fMRI	$0.51 \pm 0.02$	$0.47 \pm 0.02$	$0.47 \pm 0.02$	$0.50 \pm 0.04$	$0.49 \pm 0.04$
	Concatenation of SL	$0.76 \pm 0.02$	$0.73 \pm 0.03$	$0.80 \pm 0.01$	$0.73 \pm 0.02$	**0.76** $\pm 0.04$
	Multilayer	$0.76 \pm 0.02$	$0.78 \pm 0.01$	$0.81 \pm 0.01$	$0.75 \pm 0.02$	**0.77** $\pm 0.03$
0	Single layer DTI	$0.71 \pm 0.02$	$0.80 \pm 0.01$	$0.80 \pm 0.02$	$0.76 \pm 0.01$	**0.77** $\pm 0.04$
	Single layer GM	$0.72 \pm 0.03$	$0.63 \pm 0.02$	$0.72 \pm 0.03$	$0.60 \pm 0.03$	$0.67 \pm 0.06$
	Single layer RS-fMRI	$0.39 \pm 0.02$	$0.39 \pm 0.03$	$0.48 \pm 0.04$	$0.55 \pm 0.03$	$0.46 \pm 0.08$
	Concatenation of SL	$0.69 \pm 0.02$	$0.69 \pm 0.01$	$0.81 \pm 0.01$	$0.68 \pm 0.02$	**0.72** $\pm 0.05$
	Multilayer	$0.63 \pm 0.02$	$0.65 \pm 0.03$	$0.68 \pm 0.02$	$0.61 \pm 0.02$	$0.64 \pm 0.04$

Results indicate that the multilayer framework obtains the best results in two setups. It achieves the highest average performance (0.80) across homology dimensions 0, 1, and 2, as well as when considering only dimensions 0 and 1 with a score of 0.77. At individual level also records the best AUC-ROC value (0.83) with the Random Forest model when considering three homology dimensions. Notably, relying solely on homology dimension 0 is insufficient for the multilayer representation, whereas the single-layer DTI and the concatenation of all single layers perform comparatively well in this setting. In particular, the single-layer DTI yields robust performance, with average scores above 0.70 across all homology dimensions. Results show that the single-layer GM may provide relevant information, with performances almost reaching 0.70 and being stable at all homology dimensions. By contrast, the single-layer RS-fMRI exhibits the weakest results, with average values close to 0.50 in all cases. The concatenation of all single-layer networks achieves reasonable performance, with average scores $\ge 0.70$ across homology dimensions; however, its performance remains consistently lower than that of the multilayer framework, and in some cases even below the single-layer DTI. Taken together, these findings highlight the critical role of the DTI layer in driving the performance gains of both the multilayer framework and the concatenated single-layer networks.

**Fig. 5:**
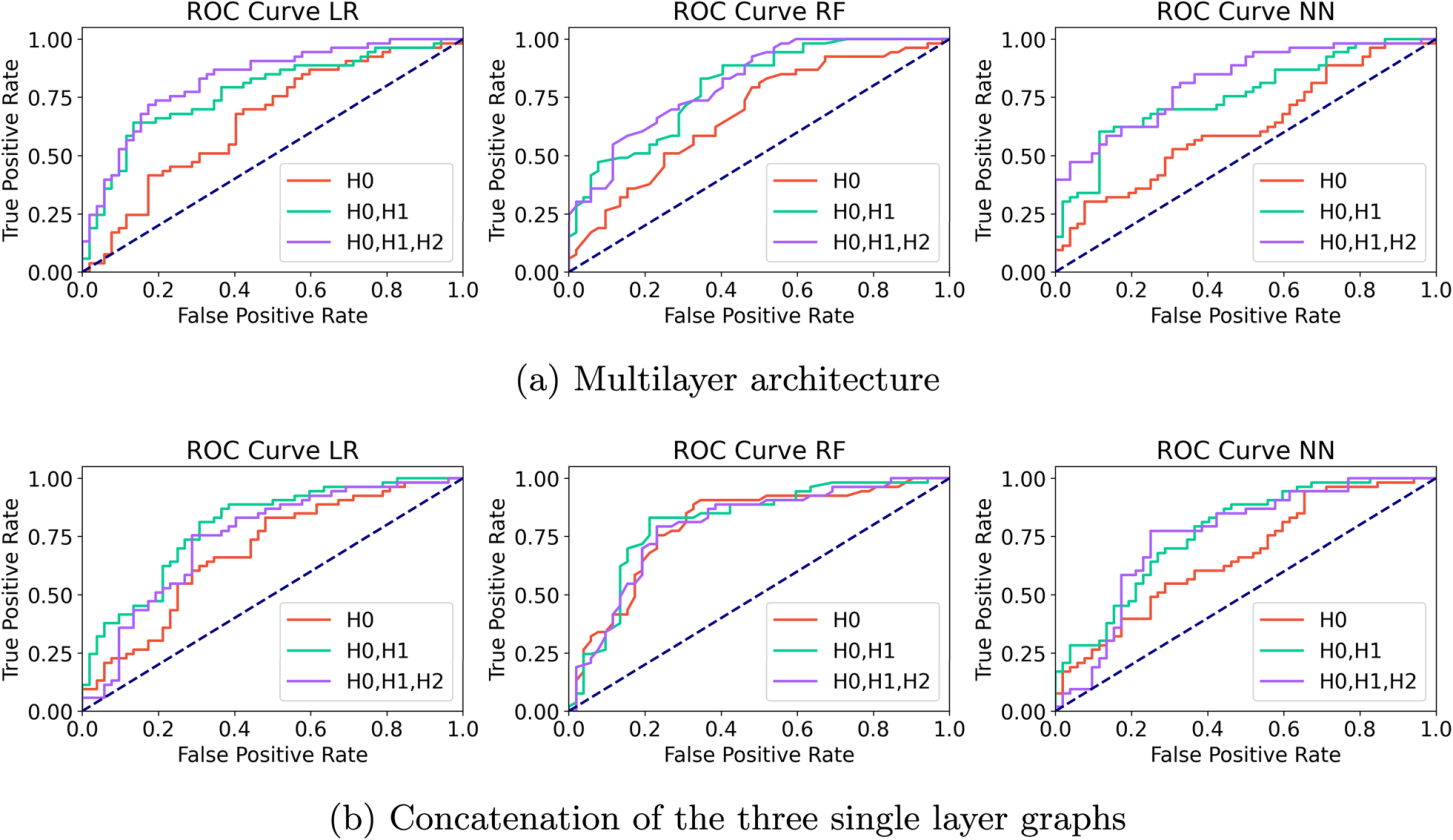
AUC-ROC evaluation comparing the multilayer architecture with the concatenation of all single layer networks for Logistic Regression (LR), Random Forest (RF) and Neural Network (NN) supervised ML models

The details of the AUC-ROC curves are shown in Figure 5, providing an in-depth view of the performance of the multilayer architecture and the concatenation of all single layer networks for Logistic Regression, Random Forest and Neural Network. As can be seen, Random Forest achieves the best results in these experiments. Notably, the multilayer architecture yields the highest accuracy, except when using only the features extracted from dimension 0. In this specific case, the AUC-ROC curve for the multilayer architecture is lower than that of the same curve obtained by concatenating all single layer features.

Table 3 reports the results obtained using our *second proposed pipeline*, in which graph-theoretical metrics are employed to generate feature representations for training supervised ML models. Apart from the feature extraction method, all other aspects of the experimental setup remain identical to those in the previous pipeline, ensuring a fair and consistent basis for comparison.

**Table 3:** AUC-ROC values (mean ± std) for different ML models applied to graph-theoretical metrics. NN denotes Neural Network, LR Logistic Regression, RF Random Forest, and SVM Support Vector Machine. The last column reports the average across all models, with bold values indicating an average performance of $\ge 0.70$. In the “Graph metric” column, LE = Local Efficiency, CC = Closeness Centrality, and BC = Betweenness Centrality. “All” denotes concatenation of all metrics into a single feature vector, while “Dim. Red.” and “Feat. Sel.” correspond to results obtained after dimensionality reduction (PCA) and feature selection (Random Forest), respectively

Graph	Network architecture	NN	LR	RF	SVM	Average
metric						
Degree	Single layer DTI	$0.64 \pm 0.01$	$0.82 \pm 0.01$	$0.82 \pm 0.02$	$0.82 \pm 0.00$	**0.78** ± 0.08
	Single layer GM	$0.58 \pm 0.01$	$0.75 \pm 0.01$	$0.70 \pm 0.02$	$0.72 \pm 0.00$	$0.69 \pm 0.07$
	Single layer RS-fMRI	$0.53 \pm 0.01$	$0.49 \pm 0.01$	$0.50 \pm 0.02$	$0.51 \pm 0.00$	$0.51 \pm 0.02$
	Concatenation of SL	$0.56 \pm 0.01$	$0.75 \pm 0.00$	$0.72 \pm 0.02$	$0.67 \pm 0.00$	$0.67 \pm 0.07$
	Multilayer	$0.54 \pm 0.01$	$0.80 \pm 0.01$	$0.81 \pm 0.03$	$0.76 \pm 0.00$	**0.74** ± 0.12
Strength	Single layer DTI	$0.49 \pm 0.01$	$0.79 \pm 0.01$	$0.81 \pm 0.02$	$0.82 \pm 0.00$	**0.73** $\pm 0.14$
	Single layer GM	$0.61 \pm 0.01$	$0.78 \pm 0.01$	$0.77 \pm 0.02$	$0.74 \pm 0.00$	**0.73** $\pm 0.07$
	Single layer RS-fMRI	$0.50 \pm 0.01$	$0.44 \pm 0.01$	$0.58 \pm 0.03$	$0.61 \pm 0.00$	$0.53 \pm 0.06$
	Concatenation of SL	$0.68 \pm 0.00$	$0.71 \pm 0.00$	$0.76 \pm 0.01$	$0.71 \pm 0.00$	**0.72** $\pm 0.03$
	Multilayer	$0.56 \pm 0.01$	$0.72 \pm 0.01$	$0.76 \pm 0.03$	$0.74 \pm 0.00$	**0.70** $\pm 0.08$
LE	Single layer DTI	$0.60 \pm 0.01$	$0.77 \pm 0.01$	$0.80 \pm 0.02$	$0.80 \pm 0.00$	**0.75** $\pm 0.08$
	Single layer GM	$0.48 \pm 0.01$	$0.78 \pm 0.01$	$0.72 \pm 0.02$	$0.76 \pm 0.00$	$0.69 \pm 0.12$
	Single layer RS-fMRI	$0.52 \pm 0.01$	$0.47 \pm 0.01$	$0.54 \pm 0.02$	$0.46 \pm 0.00$	$0.50 \pm 0.04$
	Concatenation of SL	$0.73 \pm 0.00$	$0.77 \pm 0.00$	$0.74 \pm 0.01$	$0.78 \pm 0.00$	**0.76** $\pm 0.02$
	Multilayer	$0.40 \pm 0.01$	$0.75 \pm 0.01$	$0.73 \pm 0.02$	$0.70 \pm 0.00$	$0.64 \pm 0.14$
CC	Single layer DTI	$0.66 \pm 0.01$	$0.78 \pm 0.01$	$0.80 \pm 0.02$	$0.82 \pm 0.00$	**0.78** $\pm 0.07$
	Single layer GM	$0.54 \pm 0.01$	$0.77 \pm 0.01$	$0.78 \pm 0.02$	$0.75 \pm 0.00$	**0.70** $\pm 0.09$
	Single layer RS-fMRI	$0.53 \pm 0.01$	$0.51 \pm 0.01$	$0.53 \pm 0.03$	$0.54 \pm 0.00$	$0.53 \pm 0.02$
	Concatenation of SL	$0.63 \pm 0.00$	$0.72 \pm 0.00$	$0.76 \pm 0.01$	$0.72 \pm 0.00$	**0.71** $\pm 0.05$
	Multilayer	$0.41 \pm 0.01$	$0.75 \pm 0.01$	$0.74 \pm 0.02$	$0.77 \pm 0.00$	$0.68 \pm 0.15$
BC	Single layer DTI	$0.61 \pm 0.01$	$0.68 \pm 0.01$	$0.81 \pm 0.02$	$0.78 \pm 0.00$	**0.74** $\pm 0.11$
	Single layer GM	$0.52 \pm 0.01$	$0.61 \pm 0.01$	$0.57 \pm 0.02$	$0.69 \pm 0.00$	$0.59 \pm 0.06$
	Single layer RS-fMRI	$0.50 \pm 0.01$	$0.56 \pm 0.01$	$0.66 \pm 0.04$	$0.51 \pm 0.00$	$0.54 \pm 0.04$
	Concatenation of SL	$0.68 \pm 0.00$	$0.67 \pm 0.00$	$0.81 \pm 0.02$	$0.67 \pm 0.00$	**0.72** $\pm 0.09$
	Multilayer	$0.62 \pm 0.01$	$0.63 \pm 0.01$	$0.73 \pm 0.02$	$0.70 \pm 0.00$	$0.67 \pm 0.04$
All	Single layer DTI	$0.56 \pm 0.00$	$0.55 \pm 0.00$	$0.53 \pm 0.02$	$0.52 \pm 0.00$	$0.54 \pm 0.02$
	Single layer GM	$0.51 \pm 0.00$	$0.53 \pm 0.00$	$0.63 \pm 0.02$	$0.56 \pm 0.00$	$0.56 \pm 0.05$
	Single layer RS-fMRI	$0.58 \pm 0.00$	$0.41 \pm 0.00$	$0.44 \pm 0.02$	$0.56 \pm 0.00$	$0.50 \pm 0.07$
Dim. Red.	Single layer DTI	$0.56 \pm 0.00$	$0.65 \pm 0.00$	$0.61 \pm 0.02$	$0.56 \pm 0.00$	$0.59 \pm 0.04$
	Single layer GM	$0.60 \pm 0.00$	$0.73 \pm 0.00$	$0.68 \pm 0.02$	$0.67 \pm 0.00$	$0.67 \pm 0.05$
	Single layer RS-fMRI	$0.42 \pm 0.00$	$0.43 \pm 0.00$	$0.42 \pm 0.04$	$0.47 \pm 0.00$	$0.44 \pm 0.03$
Feat. Sel.	Single layer DTI	$0.51 \pm 0.00$	$0.55 \pm 0.00$	$0.54 \pm 0.01$	$0.54 \pm 0.00$	$0.53 \pm 0.02$
	Single layer GM	$0.53 \pm 0.00$	$0.52 \pm 0.00$	$0.56 \pm 0.04$	$0.56 \pm 0.00$	$0.54 \pm 0.03$
	Single layer RS-fMRI	$0.41 \pm 0.00$	$0.43 \pm 0.00$	$0.47 \pm 0.03$	$0.54 \pm 0.00$	$0.46 \pm 0.06$

Regarding the results, we observe that the overall performance of the pipeline using graph-theoretical metrics is slightly lower than that achieved with Betti curve-based features, although it still yields competitive results. A more detailed analysis shows that the single-layer DTI network and the concatenation of all single layers perform competitively, while the multilayer architecture surpasses 0.7 only in two setups.

When it comes to individual graph metrics, strength demonstrates the highest level of performance, which yields values above 0.70 across all network architectures except the single-layer RS-fMRI. Local efficiency also performs well, particularly in the single-layer DTI, the concatenation of all single layers, and the multilayer architecture (though it performs poorly with the NN model, reaching values close to 0.40). Degree and closeness centrality also achieve good results in the multilayer and single-layer DTI settings. With respect to the classification models, Logistic Regression (LR), Random Forest (RF), and Support Vector Machines (SVM) consistently outperform the Neural Network (NN), whose suboptimal performance is likely due to the limited size of the dataset.

Finally, regarding the additional experiment in which all graph-theoretical metrics are combined into a single feature vector for each layer (creating a multi-feature configuration for each single-layer network), the results are poor for all single-layer architectures, averaging between 0.50 and 0.56. However, applying principal component analysis (PCA) for dimensionality reduction had a positive effect, mitigating noise and enhancing performance, which increased to 0.59 for the DTI and 0.67 for the GM single-layer networks. In contrast, feature selection based on importance scores derived from a Random Forest model did not yield any improvement, with results remaining close to those obtained using the complete feature vectors.

In our *third proposed pipeline*, which employs graph neural networks (GNNs) on the rich-graph representations described earlier, we evaluated 12 grid search combinations of five GNN models across five proposed rich-graph architectures (see Section 2.4.5), resulting in a total of 300 experimental configurations. To enhance the clarity and readability of the results, Table 4 reports only the best-performing result and corresponding hyperparameter combination for each GNN operator and network architecture. To ensure a fair and consistent comparison with the other pipelines, we maintain the same evaluation metrics and overall ML framework throughout the experiments.

**Table 4:** AUC ROC (mean ± std) for different operators of the GNNs, where bold values indicating an average performance of $\ge 0.70$. GCN stands for the Graph Convolutional Network, GAT for Graph Attention Network, GIN for Graph Isomorphism Network, ChebGCN for Chebyshev Graph Convolutional Neural Networks and SAGE for GraphSAGE, LR for learning rate and WD for weight decay

Operator	Network architecture	Score	Hyperparameters		
			Layers	LR	WD
GCN	Single layer DTI	$0.62 \pm 0.01$	[32,0]	$1^{-4}$	$5e^{-6}$
	Single layer GM	**0.76** $\pm 0.05$	[32,0]	$1^{-2}$	$5e^{-6}$
	Single layer RS-fMRI	$0.51 \pm 0.01$	[32,16]	$1^{-4}$	$5e^{-6}$
	Multilayer	$0.54 \pm 0.01$	[32,0]	$1^{-3}$	$5e^{-4}$
	Multimodal	**0.72** $\pm 0.02$	[32,16]	$1^{-2}$	$5e^{-6}$
GAT	Single layer DTI	**0.76** $\pm 0.02$	[32,16]	$1^{-3}$	$5e^{-4}$
	Single layer GM	**0.72** $\pm 0.06$	[32,16]	$1^{-3}$	$5e^{-6}$
	Single layer RS-fMRI	$0.51 \pm 0.02$	[32,16]	$1^{-4}$	$5e^{-6}$
	Multilayer	$0.61 \pm 0.03$	[32,0]	$1^{-3}$	$5e^{-6}$
	Multimodal	**0.78** $\pm 0.04$	[32,16]	$1^{-2}$	$5e^{-4}$
GIN	Single layer DTI	**0.76** $\pm 0.02$	[32,32,32]	$1^{-3}$	$5e^{-6}$
	Single layer GM	**0.70** $\pm 0.06$	[32,32,32]	$1^{-3}$	$5e^{-4}$
	Single layer RS-fMRI	$0.53 \pm 0.05$	[32,32,32]	$1^{-4}$	$5e^{-4}$
	Multilayer	$0.66 \pm 0.02$	[32,32,32]	$1^{-3}$	$5e^{-6}$
	Multimodal	**0.72** $\pm 0.02$	[32,32,32]	$1^{-3}$	$5e^{-6}$
ChebGCN	Single layer DTI	$0.68 \pm 0.03$	[32,32]	$1^{-2}$	$5e^{-4}$
	Single layer GM	$0.69 \pm 0.04$	[32,32]	$1^{-3}$	$5e^{-6}$
	Single layer RS-fMRI	$0.50 \pm 0.06$	[32,32]	$1^{-4}$	$5e^{-6}$
	Multilayer	$0.67 \pm 0.04$	[32,32]	$1^{-2}$	$5e^{-6}$
	Multimodal	**0.71** $\pm 0.03$	[32,32]	$1^{-2}$	$5e^{-4}$
GraphSAGE	Single layer DTI	$0.63 \pm 0.02$	[32,32,32]	$1^{-2}$	$5e^{-6}$
	Single layer GM	**0.70** $\pm 0.02$	[32,32,32]	$1^{-3}$	$5e^{-4}$
	Single layer RS-fMRI	$0.52 \pm 0.05$	[32,32,32]	$1^{-4}$	$5e^{-4}$
	Multilayer	$0.54 \pm 0.03$	[32,32,32]	$1^{-4}$	$5e^{-4}$
	Multimodal	**0.70** $\pm 0.02$	[32,32,32]	$1^{-3}$	$5e^{-4}$

As observed, the performance of the GNN-based pipeline does not surpass that of the other approaches, although several configurations yield competitive results that are comparable to those of the previous pipelines. Consistent with the behavior noted for Neural Networks in earlier experiments, we attribute this limitation in performance to the scarcity of data, which likely constrains the capacity of more complex models such as GNNs to generalize effectively. A noteworthy finding is that the DTI, GM, and multimodal (MM) graph architectures outperform the remaining configurations, achieving AUC-ROC values close to 0.75, whereas the others remain around 0.60. Among these top-performing architectures, the highest individual score is attained by the multimodal graph using a Graph Attention Network (GAT) with two attention layers, reaching an AUC-ROC of 0.78. The GCN and GIN operators also achieve strong performance, with values exceeding 0.70 across multiple network architectures. In contrast, the spectral approach implemented by ChebGCN fails to reach the 0.70 threshold in either the single-layer or multilayer architectures, although it does surpass this threshold in the multimodal setting. The GraphSAGE operator performs reasonably well in the single-layer GM and multimodal architectures but fails to capture meaningful patterns in RS-fMRI and multilayer networks, achieving scores of only 0.52 and 0.54, respectively. This limitation is likely due to the neighborhood sampling strategy, which may be less effective for brain networks that are typically small but highly interconnected.

## Discussion

In this study, we investigated the relative and combined discriminatory power of graph-theoretical and topological features extracted from multimodal brain networks. Our supervised ML approaches demonstrated three key findings: (1) DTI-based connectivity consistently yielded the most discriminative single modality features; (2) the integration of all three modalities (either via feature concatenation or a multilayer network architecture) improved classification performance relative to single modalities; and (3) within the multimodal setting, a multilayer framework that combines Betti curve–derived topological features across modalities outperformed both concatenation-based and graph-theoretical approaches in nearly all configurations (Table 2).

Our finding that DTI-derived structural connectivity matrices provide more discriminative information than GM or RS-fMRI networks aligns with prior work showing that microstructural alterations in WM tracts are often more sensitive to early or subtle pathology, particularly in neurodegenerative conditions, than macroscopic GM volume changes or functional connectivity fluctuations [77]. From a neuropathological point of view, FA metrics are sensitive to axonal integrity and myelination status [78], which can be compromised before the presence of cortical thinning or large-scale reorganizations of functional networks become measurable. In MS, demyelination and axonal loss are known to affect diffusion measures even in tissue that appears normal on conventional MRI [79]. Thus, it is not surprising that graph-theoretical metrics computed on DTI networks yielded higher classification accuracy in our unimodal experiments, using both for Betti curve–derived topological summaries and for standard network metrics. Still, it is important to emphasize that WM disruptions in MS tend to propagate along structural pathways, producing subsequent patterns of network change in other modalities that are particularly well-suited to graph-based characterization. Thus, these microstructural changes provide distinct information about connectivity integrity that is not reflected in GM atrophy or in functional connectivity measures.

While each modality contributed unique discriminative features, our results clearly show that combining modalities yielded superior performance relative to any single-modality model. This aligns with the growing consensus in network neuroscience that multimodal integration is crucial to capture the multifaceted nature of brain pathology [80]. In the multilayer approach, performance improved modestly over DTI structural connectivity, indicating that GM and functional characteristics provide valuable additional information. This suggests that MS-related changes affect complementary aspects of brain organization–structural disconnection, gray matter atrophy, and functional reorganization–which can be better captured when modalities are jointly analyzed. By integrating these diverse signals within a unified network framework, the multilayer model enhances sensitivity to subtle pathological alterations that may be missed by unimodal approaches, ultimately improving our ability to characterize, predict, and monitor disease progression.

Our empirical analysis indicates that architectures leveraging features derived from Betti curves exhibit slightly superior performance compared to those based on graph-theoretical metrics. This advantage may stem from the ability of persistent homology (PH) and Betti curves to capture higher-order topological structures in brain networks, beyond the more localized or lower-order characteristics typically represented by graph-theoretical descriptors such as node degree, shortest paths, centrality, and efficiency. Nevertheless, it is important to note that computing PH and Betti curve features is computationally more demanding. Moreover, graph-theoretical metrics offer greater interpretability and can be more readily associated with specific structural and functional properties of brain networks.

Overall, we expect that our results have several implications for understanding MS pathology and improving diagnostic or prognostic models. The dominance of DTI-based discriminability highlights the centrality of WM integrity disruptions in MS. Even when disease duration is short or lesions are clinically silent, microstructural alterations along major tracts can indicate early pathology. The multilayer, Betti-curve–driven classifier may serve as a sensitive biomarker that captures both microstructural and macroscopic network changes. Because MS pathology is heterogeneous (featuring inflammatory lesions, diffuse demyelination, and GM atrophy) a single modality will inevitably miss important aspects of the disease. Our data suggest that a multimodal topology-informed approach can better reflect this heterogeneity and potentially track progression of the disease with greater accuracy. Betti curves–especially Betti-1 and Betti-2 features–may identify “hidden” network disruptions before they become visible on conventional MRI (e.g., lesion counts or cortical volume measures). Thus, they might help stratify PwMS who are at high risk of rapid progression independent of relapse (PIRA) or at risk of conversion to progressive MS. Longitudinal follow-up studies are needed to confirm whether topological changes precede clinical deterioration or conventional imaging markers.

Beyond multiple sclerosis, we believe that this study could also support research on other neurodegenerative disorders such as Alzheimer’s disease and Parkinson’s disease. Although each of these conditions has its own characteristic distribution and involvement of brain regions, they all may manifest in changes that are detectable through graph-theoretical metrics, persistent homology, and graph neural networks. By leveraging multimodal data and topological descriptors, the most convenient analytical pipeline could be used to identify disease-specific patterns, and evaluate their discriminative power on ML algorithms. Such applications would not only test the generalizability of the methods but also contribute to the development of predictive tools for early diagnosis and monitoring across different neurodegenerative diseases.

## Limitations

Several considerations must be taken into account when interpreting the results of this study. First, the multimodal brain networks were constructed through a series of preprocessing steps–including normalization, gender and age correction, and rescaling–that, while grounded in the literature, may influence the derived topological features. Alternative preprocessing strategies could potentially enhance the sensitivity and specificity of graph-based descriptors.

Second, the pipelines employed for GM, DTI, and RS-fMRI data processing follow established state-of-the-art practices. However, many other valid approaches exist, and we did not tune or optimize parameters for our specific dataset. For instance, we excluded the cerebellum from the analysis due to susceptibility artifacts and distortions in the brainstem region in the diffusion-weighted imaging data.

Another important limitation concerns the lack of diversity in the dataset. This study relied on data from a single center, which may restrict the generalizability of the findings. Future research should aim to validate these results using larger, multicenter datasets that better capture the variability of clinical populations. However, this remains challenging due to the limited availability of multimodal data in public repositories and the lack of methodological consistency across studies.

Despite these limitations, our findings are consistent with recent studies investigating similar approaches for analyzing brain connectivity using topological and graph-theoretical techniques [39]. This supports the robustness and potential clinical relevance of the proposed methodology.

## Conclusions

This study presents a comprehensive comparison and analysis of feature extraction methods for multimodal brain networks, integrating morphological gray matter, structural connectivity (DTI), and functional connectivity (RS-fMRI) to investigate neurodegenerative diseases such as multiple sclerosis (MS). By leveraging both graph-theoretical metrics and topological data analysis through Betti curves, we systematically evaluated the effectiveness of various feature extraction techniques for characterizing individual brain networks.

Our findings demonstrate that a multilayer network architecture offers significant advantages over single layer or concatenated approaches. Specifically, this architecture more effectively preserves and represents the complex interactions among different modalities, capturing underlying mechanisms of cognitive function and neurodegeneration. These results highlight that not only the type of connectivity data matters, but also the way in which these modalities are integrated, underscoring the architectural relevance in multimodal network analysis.

Regarding future directions for this line of research, we argue that while GNNs do not currently outperform standard ML models when using either graph-theoretical metrics or Betti curve-derived features, further exploration and refinement of GNNs is essential to enhance their effectiveness. In this study, we employed standard GCN and GAT architectures; however, due to the unique characteristics of brain networks, we suggest that domain-specific operators need to be developed. These should address challenges such as the presence of highly connected nodes and the issue of graph expansion caused by the aggregation functions used in conventional GNNs. Additionally, given the limited availability of brain network datasets, we emphasize the importance of exploring the generation and use of synthetic data to support the training of GNNs and ultimately improve their performance.

## Data Availability

The code derived from this publication is publicly available (https://github.com/jcasasr/PH-BrainNets), while the data described in this manuscript are not available in the public domain. Direct requests for pseudo or fully anonymized data can be requested to the authors and will be subject to data transfer agreement stipulating the conditions for sharing and re-use.

## Footnotes

1 https://github.com/giotto-ai/giotto-tda
